# Comparing a “See-and-Treat” HPV-Based to VIA-Based Cervical Cancer Prevention Strategies Among Women Living with HIV in Cameroon: A Pilot Study

**DOI:** 10.21203/rs.3.rs-7097928/v1

**Published:** 2025-07-28

**Authors:** Simon Manga, Ye Yuanfan, Isabel Scarinci, Kathleen Nulah, Pius Tih, Juliet Ayang, Alan Tita

**Affiliations:** University of Alabama at Birmingham; University of Alabama at Birmingham; University of Alabama at Birmingham; Cameroon Baptist Convention Health Services; Cameroon Baptist Convention Health Services; Cameroon Baptist Convention Health Services; University of Alabama at Birmingham

**Keywords:** Women living with HIV, Cervical cancer screening, Human Papillomavirus, Treatment uptake, Cameroon

## Abstract

**Background:**

Women living with HIV (WLWH) have a higher vulnerability to developing cervical cancer and should be prioritized for screening. The objective of this study was to test the feasibility of integrating HPV-based vis-à-vis VIA-based cervical cancer prevention services into routine HIV Care and Treatment in Cameroon.

**Methods:**

We conducted a comparative cross-sectional study of two groups of WLWH aged 25 years and above; a group screened with Human Papillomavirus (HPV) DNA (HPV Group) and a group screened with Visual Inspection with Acetic Acid (VIA) (VIA Group), using the see-and-treat approach. The proportion of treatment-eligible women who received treatment per screening approach was calculated.

**Results:**

A total of 325 WLWH were screened as follows; VIA Group, N = 184 (45.5%) and HPV Group, N = 177 (54.5%). Among these 325 women, 74 (22.8%) were positive for either HPV and/or VIA. Of these 74, 64 (86.5%) were of the HPV Group and 10 (13.5%) were of the VIA Group. Among the 74 positive women, 14 (18.9%) received treatment. Among the 36.2% treatment-eligible women in the HPV Group, 5/64 (9.4%) had VIA-positive lesions. Among the 10 women in the VIA Group who tested positive for VIA, only 1 (10%) received treatment. Whereas, among the 5 women with VIA-positive lesions in the HPV Group, 4 (80%) received treatment.

**Conclusion:**

The treatment uptake was low (18.9%). Performing screening without treating the treatment-eligible cases will not create any reduction in cervical cancer morbidity/mortality. Therefore, more strategies are needed to enable WLWH who screen positive for HPV/VIA get adequate treatment.

## Background

Women living with HIV have up to 12-fold increased risk of developing cervical dysplasia and cancer compared to their HIV-negative counterparts [1]. Therefore, the prevalence of cervical cancer in Sub-Saharan Africa (SSA), where HIV prevalence is as high as 13–66%, [2–3] is expected to be very high. While it has been demonstrated that integration of cervical cancer prevention using visual inspection with acetic acid (VIA) into HIV care and treatment is feasible in SSA [1], it is unknown whether the integration of Human Papillomavirus (HPV)-based cervical cancer prevention is equally feasible and effective. In 2021, the World Health Organization (WHO) recommended that all cervical cancer prevention programs worldwide, including VIA-based programs, should transition to HPV-based programs immediately [4]. This is because the reduction of cervical cancer-associated morbidity and mortality is more significant with HPV-based programs compared to VIA-based programs [4]. Since setting up an HPV-based cervical cancer prevention program is costly, it is unlikely that most cervical cancer prevention programs in SSA will transition to HPV-based programs in the foreseeable future as this transition will likely occur gradually over time. It is, therefore, necessary to evaluate the feasibility of HPV-based cervical cancer prevention in SSA to inform this eminent transition.

WHO has proposed two HPV screening approaches for women aged 30 and over (or 25 and over for those living with HIV): the “HPV see-and-treat approach”, and the “HPV see-triage-and-treat approach.” For the HPV “see-and-treat” approach, all women positive for oncogenic HPV are treated, but VIA/colposcopy is first conducted to determine the presence or absence of a precancerous/cancerous lesion. Those with visible lesions are treated depending on the characteristics of the lesion. Those without any visible lesions on VIA are treated with ablative treatment. For the HPV “see-triage-and-treat” approach, every positive HPV test is triaged with another test, VIA/colposcopy or cytology, and the treatment depends on the results of the triage test: if the triage test is positive, she is treated; otherwise, she is not treated [2].

Three models of integrating cervical cancer prevention into HIV care and treatment have been described: 1) integration within the same HIV clinic through capacity building of the existing staff, 2) integration through colocation of the two services within the same institution, and 3) integration through multifaceted coordination across the care pathway [5]. Each model has its unique advantages and disadvantages. The model of integration within the same clinic is associated with limited loss to follow-up compared to the other models but it is more costly to implement [5].

With the advent of antiretroviral treatment (ART), women living with HIV are now living longer, increasing their overall risk of cervical cancer [6]. Failure to integrate cervical cancer prevention into HIV care and treatment services would be a big disservice to the great accomplishments that have been made in the ght against HIV [7].

The overarching objective of this study was to test the feasibility of integrating HPV testing into HIV Care and Treatment with focus on treatment uptake of eligible women. This was done through comparing the integration of a “see-and-treat” HPV-based and a “see-and-treat” VIA-based strategy into routine HIV care for women living with HIV. Prioritizing cervical cancer screening among women living with HIV has the potential to significantly reduce cervical cancer-associated morbidity and mortality among these vulnerable women who are most at risk for cervical cancer, and this is in line with the WHO cervical cancer elimination strategy by 2030. Our central hypothesis was that, treatment uptake among treatment eligible women will be similar in both approaches; the VIA-based approach and the HPV-based approach.

## Materials and Methods

We conducted a comparative cross-sectional study of two convenient groups of women living with HIV; a group screened primarily by the Human Papillomavirus DNA (HPV Group) and a group screened primarily by Visual Inspection with Acetic Acid (VIA) (VIA Group), using the see-and-treat approach. The study received Institutional Review Board (IRB) approval from the Cameroon Baptist Convention Health Services (CBCHS) # IRB2022–63 and the University of Alabama at Birmingham (UAB) # IRB-300009970. Participants signed an informed consent, and the data collected complied with relevant patient data protection rules and guidelines. This research was conducted in compliance with the Helsinki Declaration.

### The Setting

The study was conducted at the Nkwen Baptist Hospital (NBH), one of the largest institutions of the CBCHS located in Bamenda, the Northwest Region of Cameroon. NBH has an enrollment of 4,000 persons on antiretroviral treatment (ART), of whom over 60% are women.

The CBCHS is a large faith-based healthcare organization with a network of 95 health facilities in 8 of the 10 regions of Cameroon. The CBCHS runs a large HIV Care and Treatment program with funding from CDC/PEPFAR in three of the 10 regions of Cameroon. The CBCHS currently supports over 95,000 people on ART in 80 health facilities. The CBCHS also runs the largest cervical cancer prevention program in Cameroon, called the Women’s Health Program (WHP). WHP has screened over 150,000 women for cervical cancer. The program started in 2007 as a VIA-based program using Visual Inspection of Acetic Acid and Lugol’s Iodine (VIA-VILI) enhanced by digital cervicography (DC) [8]. In DC, a camera is used to take real-time, highly-magnified images of the cervix projected onto a TV monitor so that both the woman and the provider can see the cervix’s life at the same time, mimicking video colposcopy [9]. In 2020, WHP added HPV testing with self-collected samples in some of its sites. Therefore, WHP runs HPV-based program in some of its sites that have access to HPV testing while the other sites continue to use the VIA-based approach.

Among the three models of integrating cervical cancer prevention into HIV Care, in this study, we employed the model of integration through the colocation of the two services within the same institution. This is because NBH already has a well-established WHP clinic that renders comprehensive cervical cancer screening and prevention services. This facilitated bi-directional referrals of these women between the two clinics: the HIV Care Clinic and the Cervical Cancer Screening Clinic.

### The Procedure

Women living with HIV and receiving HIV care at NBH, aged 25 years and above, were prospectively classified into two non-randomized groups based on the type of screening test. One group was screened using HPV DNA (the HPV Group), and the other group was screened using VIA-DC (the VIA Group). A referral card was developed that was used to refer the eligible women from the HIV Care and Treatment Clinic to the WHP Clinic. The women who were enrolled in the first half of the study were assigned to the HPV Group while those enrolled in second half of the study were assigned to the VIA Group. The entire study ran from January to September 2023.

In 2020, WHP added HPV testing with self-collected samples using AmpFire HPV testing for women aged 30 years and above (or 25 years and above if they are HIV positive) in some of its sites. The AmpFire HPV analyzer, Atila BioSystems, Sunnyvale, CA, USA, is a relatively new HPV detection technology that uses a multiplex isothermal real-time fluorescent detection assay and can detect 14 high-risk HPV types while genotyping types 16 and 18 simultaneously. That is, the results are presented in three panels: the HPV 16 panel, the HPV 18 panel, and a combination of the other 12 HPV types. The main advantage of AmpFire is that HPV can be detected directly from raw samples like dry swab samples, and the time from sample to result is just an hour. In addition, it does not require batching and can be easily performed in a clinical setting [10].

The HPV Group: Specimens for HPV testing was self-collected per WHP guidelines and handed to the WHP clinic staff. The specimens were appropriately labeled by the clinic staff and shipped to a WHP facility in Yaoundé for analysis. The results were emailed to the project staff at NBH within a week. Patients were then called and informed of the availability of their results. Women positive for HPV were further screened with VIA-DC. Those positive for the VIA-DC were treated depending on the characteristics of the lesion and the treatment was offered on the same day.

VIA Group: Those in the VIA group were screened only with VIA-DC per the program guidelines and same day treatment was offered to those positive for VIA.

Treatment: In both Groups, the treatment decision was based on VIA-DC results. Precancerous lesions of the cervix that met cryotherapy eligibility were provided thermal ablation (TA) while lesions that met eligibility for large loop excision of the transformation zone (LLETZ) were provided LLETZ. Those needing TA were provided same-day treatment, while those needing LLETZ were scheduled for treatment within a month. A biopsy was performed on lesions suspicious of invasive cervical cancer (ICC), and if the histopathology confirmed the ICC, the patient was referred to a specialized center for appropriate treatment. For the HPV Group, TA was provided to all those positive for HPV and had no lesion on VIA-DC.

Treatment uptake was used to measure the success in both Groups. That is, to measure success, we used the rate of treatment uptake among treatment eligible women in the Group.

### Data Analysis Plan

Chi-square tests were used to compare participants’ categorical characteristics, and t-tests and Fisher’s exact tests (as needed) were used to compare participants’ continuous variables by VIA –Group and HPV Group. Variables with p < 0.05 in the bivariate analysis were included in the multivariable analyses. The proportions of women who received treatment per Group were also calculated and compared between them. We did not perform logistic regression because of the very small sample size of treatment eligible women.

## Results

A total of 325 women living with HIV were screened as follows: VIA Group, N= 184 (45.5%) and HPV Group, N= 177 (54.5%) (Table 1). Among the 325 women screened, 74 (22.77%) were positive for either HPV and/or VIA. Of these 74, 64 (86.5%) were of the HPV Group and 10 (13.5%) were of the VIA Group.

In the HPV Group, the prevalence of HPV Types was as follows: HPV 18: 3/64=4.7%, HPV 16: 12/64=18.8%, other HPV types, 51/64=79.7% (patients could be co-infected with multiple HPV types).

Table 1 shows the participants’ characteristics according to VIA Group and HPV Group. Viral load was the only variable that was statistically significant among the two Groups (p = 0.001).

Of the 74 women eligible for treatment, only 14 (18.9%) received treatment while 60 (81.1%) did not receive treatment. According to Table 2, there were more treatment-eligible women in the HPV Group than in the VIA Group.

**Figure 1:**
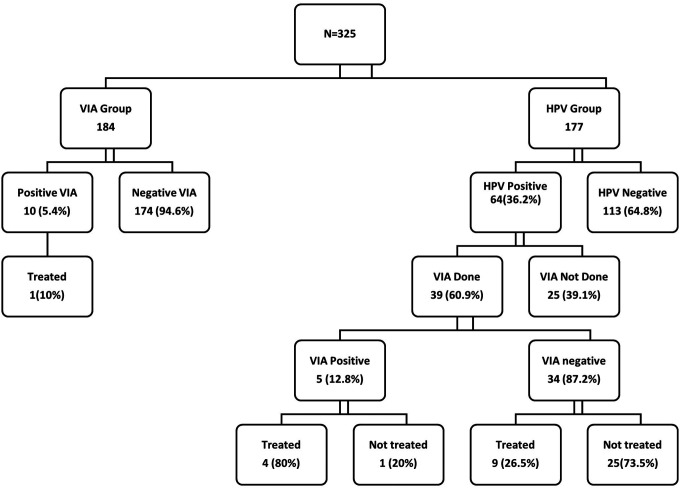
Flow Diagram Activities.

Women less than 50 years were more likely to be positive for VIA compared to those over 50 (p = 0.036). Among the 10 women in the VIA Group who tested positive for VIA, only 1 (10%) received treatment. Whereas, among the 5 women with VIA-positive lesions in the HPV Group, 4 (80%) received treatment (Figure 1). Women who were positive for both HPV and VIA were more likely to accept treatment compared to those who were positive just for VIA or HPV.

In this study, A total of 36.2% of participants in the HPV Group had treatment eligibility compared to only 5.4% in the VIA Group (p<0.001). Among the 36.2% treatment-eligible women in the HPV Group, 5/64 (9.4%) had VIA-positive lesions.

We did not run logistic regression for predictors associated with treatment uptake because of the very small sample of treatment eligible women in the VIA Group.

## Discussion

Our study piloted the integration of a “See and Treat” HPV-based cervical cancer prevention program vis-à-vis VIA-based cervical cancer prevention, taking into account the collocation of an HIV Care and Treatment Center and a cervical cancer prevention program clinic in the same hospital [11]. The overall treatment uptake was low (18.1%), signifying poor success. In our previous work in Cameroon, we found treatment uptake of 55.9% in the general population [12]. In a Namibian study, treatment uptake was as high as 82% in the general population [13]. The very low treatment uptake among women living with HIV (WLWH) is very concerning and calls for further investigation. WLWH have potentials for higher treatment rates because of their frequent visits to the hospital compared to their HIV negative counterparts. A number of the women in our cohort indicated the inability to adhere to the one-month mandatory period of abstinence post-treatment. There is a need for further studies to examine the reasons for their reluctance in the one-month abstinence. In our previous work in Cameroon, we discovered a very low treatment uptake among Female Sex Workers (FSWs), and this was related to the fact that they earned their living through sex work [14]. Since the prevalence of HIV among FSWs is higher than that of the general population, it could be that the women in our study were coincidentally FSWs. Unfortunately, we did not collect sex work status in this study.

Contrary to our findings, a Nigerian study that piloted the HPV-based see and treat approach into HIV Care and Treatment found a treatment uptake of up to 95% [15]. However, this study was limited by a small sample size of 98 women. In addition, the authors stated that their participants for the study were purposefully selected. The reasons why they did a purposeful sampling in a quantitative study were not explained. This might have introduced a bias in their study because they might have selected only participants who were likely to accept treatment. One advantage of this Nigerian study is that their HPV analyses were done on the spot, unlike in our study where they were shipped to a faraway Region for analysis. Another major difference between our study and the Nigerian study is that we piloted the model of colocation of the HIV clinic and cervical cancer prevention clinic in the same hospital, while in Nigeria, they piloted the integration of cervical cancer prevention within the same HIV clinic. However, their report did not specify if they used the same staff in the HIV clinic for cervical cancer screening or if the staff were brought in from another unit.

Surprisingly, the treatment uptake in the HPV Group was higher than that of the VIA Group. One would have expected higher treatment rates in the VIA Group because the VIA results were available on the spot and treatment was offered on the spot. Contrary to the VIA Group, participants in the HPV Group had to provide a specimen for HPV testing, wait for the results in a week, and then the positives had to undergo VIA-DC before treatment was offered. The reason for the poorer treatment uptake in the VIA Group is unclear.

Just like the Nigerian study, a Kenyan study has reported that integrating cervical cancer screening into HIV Care and Treatment is very feasible in SSA. This study reported a feasibility rate of 64.5% [16]. However, the study did not use treatment uptake as a measurement for successful integration. A successful cervical cancer prevention program cannot be measured by the number of women screened but by the number of treatment-eligible women who receive treatment [12, 17]. Screening women for cervical cancer without appropriate treatment of the treatment eligible patients cannot contribute in the elimination of cervical cancer. In our study, the women accepted screening in their hundreds, but we will be doing a disservice to conclude that this was a success without taking into consideration the treatment uptake.

In our study, the prevalence of HPV 16 and 18 was lower than that of the other 12 HPV types present in the AmpFire platform. HPV 18 had the lowest prevalence. This is consistent with our previous work whereby we performed HPV genotyping among 600 FSWs in Cameroon. In this study, HPV 18 had the lowest prevalence of 2.8% followed by HPV 16 with 5.2%. HPV 51 had the highest prevalence (14%), followed by HPV 53 (12.4%) and HPV 52 (12.2%) [17]. According to current literature, HPV 16 and 18 are the most prevalent oncogenic types worldwide [18–19]. This appears to be the reverse in Cameroon. Larger prospective studies are required to examine this phenomenon.

The major strength of our study is that it is among the first studies to pilot the integration of HPV-based cervical cancer prevention vis-à-vis VIA-based cervical cancer prevention into HIV Care and Treatment. In addition, we piloted the integration based on collocation between the HIV care clinic and the cervical cancer prevention clinic in the same hospital. So, our study environment was the ideal working environment. Moreover, our success of integration was measured by treatment uptake for treatment-eligible patients.

The study had some weaknesses, which include: 1. The women were not randomly assigned into the two groups, 2. The HPV analyzer was not available in the study hospital, and specimens had to be shipped to a hospital in a faraway region for analysis. This contributed to prolonging results time, thus affecting triage and treatment uptake in the HPV Group, 3. The sample size of the treatment-eligible women of the VIA Group was very small, and this made it somewhat difficult to compare treatment uptake between the two groups adequately.

## Conclusion

The overall treatment uptake among treatment-eligible women was low. A successful cervical cancer prevention integration into HIV Care and Treatment has to be associated with high treatment uptake among the treatment-eligible patients. The reasons for the poor treatment uptake among treatment-eligible WLWH has to be carefully studied in order to propose strategies that can overcome the identified barriers.

## Figures and Tables

**Table 1.** Participants’ characteristics according to VIA Group and HPV Group (N=325)

Characteristics	VIA Group	HPV group (with HPV results)	P Value
	N=148 (45.5%, 95%CI: 40.1%−51.0%)	N=177 (54.5%, 95%CI: 49.1%−59.9%)	
**Age**			0.107
**<50 yrs**	86 (52.7)	109 (61.6)	
**≥50 yrs**	70 (47.3)	68 (38.4)	
Marital Status *miss 8			0.052
Married	55 (37.9)	83 (48.5)	
Others	90 (62.1)	88 (51.5)	
Educational Level *miss 4			0.108
<8 yrs	90 (60.8)	120 (69.4)	
≥ 8 yrs	58 (39.2)	53 (30.6)	
Occupation *miss2			0.085
Domestic Worker/housewife/farmer	60 (40.8)	89 (50.6)	
Trader/business	39 (26.5)	48 (27.3)	
Others	48 (32.7)	39 (22.2)	
**Viral load N=305**			0.001
<40	127 (88.2)	158 (98.1)	

**Table 2:** Participants’ Characteristics According to Treatment Eligibility

	Positive		P value
	VIA Group	HPV Group	
	N=10 (13.5%, CI: 5.7%−21.3%)	N=64 86.5%, CI: 78.7%−94.3%)	
**Age**			0.036
**<50 yrs**	9 (90.0)	32 (50.0)	
**≥50 yrs**	1 (10.0)	32 (50.0)	
Marital Status *miss 5			0.285
Married	6 (66.7)	26 (43.3)	
Others	3 (33.3)	34 (56.70	
Educational Level *miss3			1.000
<8 yrs	7 (70.0)	43 (70.5)	
≥ 8 yrs	3 (30.0)	18 (29.5)	
Occupation *miss1			0.498
Domestic Worker/housewife/farmer	3 (30.)	31 (49.2)	
Trader/business	4 (40.0)	19 (30.2)	
Others	3 (30.0)	13 (20.6)	
Viral load *miss5			0.053
<40	8 (80.0)	58 (98.3)	
ARVs Treatment Regimen *miss 2			1.000
8A	8 (80.0)	50 (80.7)	
Others	2 (20.0)	12 (19.3)	
*Treated	1 (10.0)	13 (20.3)	0.676
**VIA Positive Treated	1 (10.0)	4 (30.8)	0.357

* A total of 74 women were eligible for treatment and 14 received treatment

** A total of 14 patients received treatment; 1 from the VIA group and 4 from the HPV group

## Data Availability

All data generated or analyzed for this study have been included in this published article
